# A Systematic Review of miRNA and cfDNA as Potential Biomarkers for Liquid Biopsy in Myocarditis and Inflammatory Dilated Cardiomyopathy

**DOI:** 10.3390/biom12101476

**Published:** 2022-10-13

**Authors:** Piotr Lewandowski, Marcin Goławski, Maciej Baron, Edyta Reichman-Warmusz, Romuald Wojnicz

**Affiliations:** 1 Department of Histology and Cell Pathology, Faculty of Medical Sciences in Zabrze, Medical University of Silesia, 40-055 Katowice, Poland; 2 Department of Pharmacology, Faculty of Medical Sciences in Zabrze, Medical University of Silesia, 40-055 Katowice, Poland; 3 Silesian Nanomicroscopy Center, Silesia LabMed—Research and Implementation Center, Medical University of Silesia, 40-055 Katowice, Poland

**Keywords:** liquid biopsy, myocarditis, dilated cardiomyopathy, microRNA, circulating free DNA

## Abstract

Myocarditis and inflammatory dilated cardiomyopathy are cardiac diseases leading to heart failure. Liquid biopsy is a concept of replacing traditional biopsy with specialized blood tests. The study aim was to summarize and assess the usefulness of microRNAs and circulating free DNA as biomarkers of myocardial inflammation. For this systematic review, we searched Scopus, Embase, Web of Science, and PubMed. All studies measuring microRNAs in serum/plasma/cardiac tissue or circulating free DNA during myocarditis and non-ischemic dilated cardiomyopathy in humans in which healthy subjects or another cardiac disease served as a comparator were included. Data were extracted and miRNAs were screened and assessed using a scale created in-house. Then, highly graded miRNAs were assessed for usability as liquid biopsy biomarkers. Of 1185 records identified, 56 were eligible and 187 miRNAs were found. We did not identify any studies measuring circulating free DNA. In total, 24 of the screened miRNAs were included in the final assessment, 3 of which were selected as the best and 3 as potential candidates. We were not able to assess the risk of bias and the final inclusion decision was made by consensus. Serum levels of three miRNAs—miR-Chr8:96, miR-155, and miR-206—are the best candidates for myocardial inflammation liquid biopsy panel. Further studies are necessary to prove their role, specificity, and sensitivity.

## 1. Introduction

Myocarditis (MCI) is a form of severe heart disease with progressive cardiomyocyte damage often leading to cardiac dysfunction, ventricular remodeling, and the development of inflammatory dilated cardiomyopathy (InfDCM). MCI is predominantly caused by viral infections. Those infections, in turn, lead to the development of virus-associated autoimmune MCI [1]. Early diagnosis, particularly in active MCI, is crucial for providing optimal treatment and suppression of disease progression. According to the current European Heart Society (ESC) guidelines, MCI can be suspected based on clinical history, signs and symptoms, ECG, laboratory tests, echocardiography, or cardiac magnetic resonance. Nevertheless, endomyocardial biopsy (EMB) remains the “gold standard” for MCI diagnosis [2]. In accordance with current guidelines, an endomyocardial biopsy (EMB) should be performed when there is no possibility of differentiating the cause of DCM with other methods [2,3].

**Figure 1:**
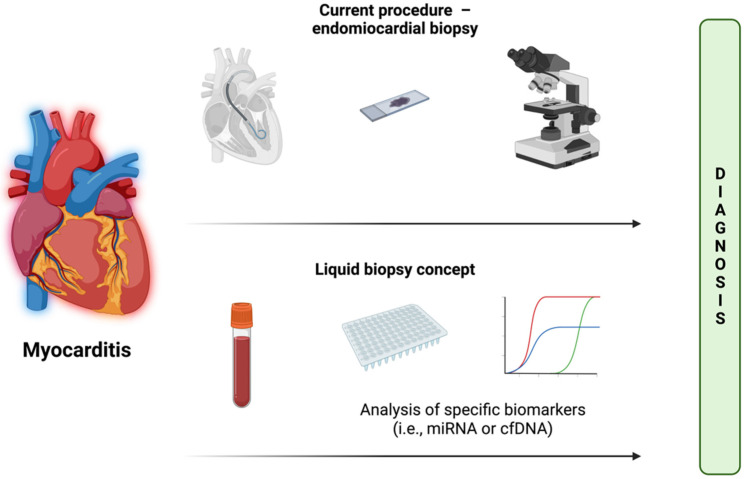
Concept of liquid biopsy. In comparison to endomyocardial biopsy, it requires only a blood sample. The goal is to achieve comparable diagnosis accuracy. Created with BioRender.com. Accessed on 7 September 2022.

Liquid biopsy (LB) is the analysis of non-solid biological tissue, primarily blood, for novel disease-specific biomarkers, and can provide accuracy comparable to classical biopsy [4]. Liquid biopsy is potentially a novel, less-invasive method for MCI detection. The main obstacle in the development of LB is finding optimal (sensitive and specific) biomarkers for inflammatory myocardial diseases. Two types of nucleic acids: microRNA (miRNA) and circulating free DNA (cfDNA), which circulates freely in the blood, have the potential to be such biomarkers [5]. A schematic representation of the liquid biopsy concept is shown in Figure 1.

Micro RNAs are short (~22 bases) non-coding RNAs. They are transcribed from DNA and processed into mature miRNA in a few steps. The main role of miRNA regulation of gene expression is through interactions with mRNAs. The miRNA expression changes dynamically, maintaining cell homoeostasis, especially in response to changes in the cellular environment. Micro RNA can be released into various bodily fluids, and, thanks to its stability and faster responses in its levels in comparison to proteins, miRNA has the potential to become a novel, disease-specific biomarker.

CfDNA are 50–200 bases long DNA fragments released into circulation from the nuclei and mitochondria of cells undergoing necrosis and apoptosis [6].

The main purpose of this systematic review is to summarize current studies on miRNA or cfDNA measurements and assess their usefulness as LB markers for the detection of myocardial inflammation.

## 2. Materials and Methods

### 2.1. Search Strategy

Four databases—Scopus, Embase, Web of Science and PubMed—were searched to identify eligible studies through 18 July 2022. The search was performed using the keywords listed in Supplementary Materials S1. The results were merged, and automatic and manual duplicate removal was applied. Articles found outside the search (i.e., in the references of the articles included in the search) were also included.

Each record identified was screened by title and abstract (P.L. and M.B.) followed by full-text screening (P.L. and M.G.). The inclusion and exclusion criteria are summarized in Table 1 and Table 2, respectively.

This systematic review was performed in accordance with Preferred Reporting Items for Systematic Reviews and Meta-Analyses (PRISMA) guidelines [7].

### 2.2. Data Extraction

Data were extracted from all included studies. The extracted data included a list of assessed miRNAs, number of subjects, subject age (mean and standard deviation), % of female subjects, sample type, miRNA screening method (if applicable), miRNA isolation method, measurement method, reference gene (if applicable), clinical diagnosis, and diagnostic criteria of each study group. In the case of high-throughput methods, such as miRNAseq or microarray, only miRNAs described as significantly dysregulated were extracted. In the studies involving screening and proof measurements, only the proof measurements were extracted into the database.

### 2.3. Disease Classification

For the purposes of analysis, study groups were classified into one of three diseases: myocarditis, inflammatory dilated cardiomyopathy, and non-ischemic idiopathic dilated cardiomyopathy. The criteria for each study group classification are provided in Table 3. If the disease could not be clearly classified with our criteria, the final decision was based on the consensus of three researchers (P.L., M.G., and R.W.). Studies that did not include a population classifiable with these criteria were excluded. Diagnosis of DCM was assumed to be appropriate, even when authors did not describe the diagnosis methodology.

### 2.4. miRNA Screening

The list of all miRNAs included was extracted. MicroRNA-5p and microRNA-3p of the same origin were assessed together. Each miRNA was independently assessed for utility as a liquid biopsy biomarker for myocarditis/InfDCM diagnosis by two researchers (P.L. and M.G.). The assessment was performed in accordance with the scale presented in Table 4. Micro RNA assessed by at least one researcher as 4 or 5 was included in the final assessment.

### 2.5. Final Assessment

The final miRNAs evaluation was performed by the analysis of the complete evidence—the available dataset (including results from miRNA screening—miRNAseq, microarrays, etc.), and the results of an article search in Google Scholar and PubMed for each miRNA. A miRNA was considered relevant if the results were strong and consistent and no serious evidence for non-specificity was found. The assessment was performed by two researchers (P.L. and M.G) and the final decision was made by consensus.

## 3. Results

### 3.1. Identification and Screening Results

**Figure 2:**
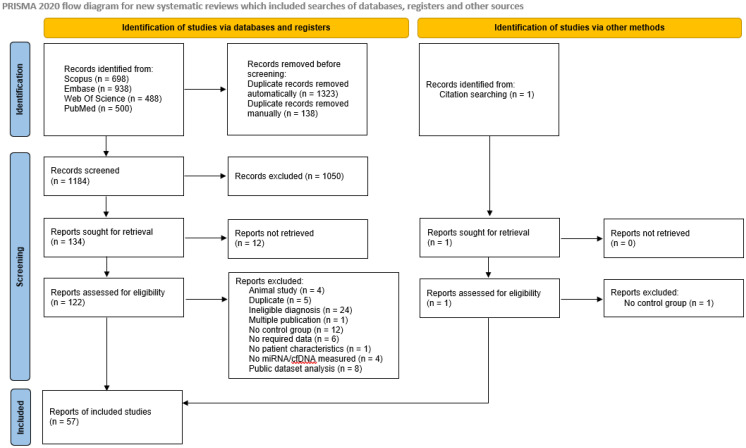
PRISMA flowchart of studies screened and included in this review (http://www.prisma-statement.org/; Accessed on 5 September 2022).

A total of 2624 records were retrieved from the Scopus, Embase, Web of Science, and PubMed Databases. After duplicate removal, 1185 articles identified in the databases and 1 article found outside of the search results were screened by title/abstract and 122 of them were screened by full text. In total, 55 studies were included. A flowchart is presented in Figure 2. A complete list of excluded studies with exclusion reasons is presented in Tables S1 and S2.

### 3.2. Characteristics of Included Studies

Data regarding 4577 patients were extracted from the 55 included studies. In 23 studies, miRNA levels were measured during MCI, in 4 during InfDCM, and in 28 during Ni-IDCM. None of the studies assessed cfDNA in MCI/InfDCM. Healthy subjects were most often used as a control (*n* = 51). The diagnosis was verified with EMB in only 8 studies (available in Table S3). In 18 studies measurements were preceded with screening by a high-throughput method (microarray: 8 studies; miRNAseq: 4 studies; qRT-PCR: 4 studies; bead-based method: 1 study; and murine experiment with microarray: 1 study). Overall, 32 out of 51 studies provided information about PCR reagents and conditions. However, many of them did not fulfill MIQE guidelines for qRT-PCR reporting [8]. Data regarding all the included studies are available in Table S3.

### 3.3. Results of miRNA Screening and Assessment

A total of 187 miRNAs were screened. Micro RNAs graded high (4) or very high (5) were assessed for final inclusion. The results of the screening and assessment are summarized in Table 5. Short descriptions of reasons for the exclusion of assessed miRNA are available in Supplementary Materials Table S4.

## 4. Discussion

### 4.1. Best Candidates for Liquid Biopsy Biomarkers

During miRNA assessment, three miRNAs were selected as liquid biopsy markers for the diagnosis of MCI/InfDCM. The selection was based on the overall available evidence, including homogeneity of the results, unambiguous dysregulation of miRNA expression, specificity, and sensitivity, as well as knowledge of the release mechanisms.

#### 4.1.1. miR-Chr8:96

**Evidence**: MiR-Chr8:96 as a myocarditis biomarker has been validated in only one study, but this was a high-quality study. This study involved the screening of T cells from mice with myocarditis and myocardial infarction with miRNA microarrays, selecting the most overexpressed and specific miRNA in myocarditis and validation in mice plasma. Then, a human homolog was selected and validated in plasma samples of five large patient cohorts involving not only patients with myocarditis and healthy subjects, but also patients diagnosed with ST elevated myocardial infarction (STEMI), non-ST elevated myocardial infarction (NSTEMI), myocardial infarction with nonobstructive coronary arteries (MINOCA), and Th17 dependent diseases (rheumatoid arthritis, spondylarthritis, psoriasis, and multiple sclerosis). MiR-Chr8:96 serum levels allowed patients with myocarditis to be distinguished from healthy donors and cases of myocardial infarction with high sensitivity and specificity (receiver operating characteristic—area under curve (ROC-AUC): 0.988, 0.927 for differentiation of MCI from healthy and myocardial infarction patients, respectively) [9].

**Role and source:** MiR-Chr8:96 is novel, and its exact role is unknown. The role of the murine homolog—mmu-miR-721 is also unknown. Data from the presented study suggest that it is expressed in Th17 cells during myocarditis [9].

**Discussion:** There is robust evidence for the specificity and selectivity of this miRNA. However, some limitations should be underlined. The aforementioned study did not involve confirmation of myocarditis using EMB. In addition, CMR was not performed in most cases. The role of miR-Chr8:96 in Th17-dependent diseases is not presented. Moreover, the study did not include DCM patients [9].

#### 4.1.2. miR-155

**Evidence**: MiR-155 level was measured in 13 studies [10,11,12,13,14,15,16,17,18,19,20,21,22]. Its overexpression was found mainly in MCI (5 studies) [10,13,14,19,21] and InfDCM (3 studies) [12,18,20]. Only one study identified increased expression of miR-155 in ischemic cardiomyopathy [20]. The remaining studies did not detect increased miR-155 expression in other myocardial diseases (non-inflammatory DCM [11,12,17,18,19], rheumatic carditis [15], or hypertrophic cardiomyopathy [20]). Two studies performed ROC-AUC analysis, one for distinguishing between MCI and healthy subjects (AUC = 0.901) [13], and one for InfDCM and healthy subjects (AUC = 0.68) [12]. One study proved the ability of the levels of miR-155 to differentiate between Coxsackie Virus B3 positive and negative DCM [21]. Additionally, studies where MiR-155 expression was increased, exhibited homogeneity in the results between different types of samples: plasma [10,12], serum exosomes [13] and biopsy samples [14,18,19,21]. One study did not identify statistically significant differences between miR-155 expression in viral myocarditis and healthy subjects [22]. One study identified decreased expression in non-viral idiopathic DCM [10], and one identified a decrease in expression of acute viral myocarditis [16].

**Role and source:** The role of miR-155 is quite well understood in comparison to other miRNAs. It is expressed in activated T cells, B cells, monocytes, and macrophages [23]. MiR-155 takes part in the immune response by promoting inflammation through the proliferation of macrophages, NK cells, and B and T cells, stimulating IL-6, TNF-alpha, and IFN-gamma synthesis, and other proinflammatory processes [24]. Corsten et al. proved that in MCI miR-155 expression in heart tissue is localized mainly in macrophages and T cells [14]. In another study, the miR-155 expression correlated with the number of CD68-positive cells (macrophages) during myocarditis [19]. The inhibition of miR-155 in mice resulted in the reduced expression of proinflammatory cytokines (IL-6, TNF-alpha, IFN-gamma, and IFN-beta) and decreased T cell numbers in the myocardium [14].

**Discussion:** The decision to include miR-155 was not straightforward. Key arguments in its favor were the high number of studies, known role/source, homogeneity between serum/tissue samples, and strong correlation with inflammatory infiltration in the myocardium. On the other hand, miR-155 is not MCI/InfDCM-specific as reported by some studies. Moreover, it was proved that miR-155 is also overexpressed in myocardial infarction, especially in its late phase [25]. The expression of miR-155 is related to the activity of immune cells. This may be an advantage of miR-155 because it reflects the current immune response in the myocardium. It could be measured easier and faster than EMB. Interestingly, Zhang et al. showed the high suitability of miR-155 for the early detection of fulminant MCI [13].

#### 4.1.3. miR-206

**Evidence**: miR-206 was evaluated in four studies [12,18,19,26]. Two of them identified its overexpression in InfDCM vs. non-inflammatory DCM [12,18], one in myocarditis vs. healthy subjects [19] and one in Ni-IDCM vs. healthy controls [26]. One study performed a ROC curve analysis (AUC = 0.607) [12]. MiR-206 was measured in different sample types: plasma [12], CD4^+^ T cells [26], and EMBs [18,19]. The results were similar regardless of sample type.

**Role and source:** The role of miR-206 is multifaceted and probably not fully understood. The current evidence suggests a role in muscle differentiation, regeneration, hypertrophy, and angiogenesis [27]. Kleeberger et al. proved that the concentration of miR-206 is inversely correlated with left ventricular ejection fraction in patients undergoing transcatheter aortic valve implementation (TAVI). They suggested that miR-206 is overexpressed in conditions of heart failure. However, as the authors observed, the miR-206 level remains stable after TAVI, whereas classical biomarkers of myocardial damage (troponin, creatinine kinase-MB) rise [28]. MiR-206 expression may not be related to myocardial damage, but it can play a role in the process leading to cardiomyocyte apoptosis [27]. MiR-206 is essential in cardiac hypertrophy during pressure-overload, but also protects cardiomyocytes from apoptosis [29,30,31]. Additionally, miR-206 suppresses the expression of connexin-43, and its upregulation may promote arrhythmias [32]. Moreover, mir-206 also impacts T-cell differentiation, suggesting a role in mediating inflammation [33].

The origin of circulating miR-206 remains unknown [12,27]. Two studies proved it is present at increased levels in myocardial tissue during MCI [19] and InfDCM [18]. On the other hand, Obradovic et al. suggested that it may be linked with systematic myositis [12] considering the abundance of miR-206 in all types of muscle cells [27].

**Discussion:** MiR-206 is a potential candidate for becoming a biomarker for the differentiation of inflammatory and non-inflammatory cardiac diseases. Its role may be interesting because it is not secreted during direct myocardial injury, but instead in other poorly-defined conditions [27]. Due to this, further studies are necessary to better understand its role and function.

### 4.2. Potential Candidates for Liquid Biopsy Biomarkers

Other miRNAs did not fulfill our inclusion criteria for the LB panel. The results of studies regarding some miRNAs were inconsistent. These miRNAs correlated with myocardial injury and/or fibrosis but not inflammation. However, a few were particularly interesting and unique. Accordingly, three are discussed here—miR-30a, miR-145a, and miR-145b.

MiR-30a is a miRNA probably linked with the immune response during Coxsackie infection [34]. Two studies showed its overexpression in MCI [13,16], but one showed an increase in both InfDCM and Ni-IDCM (much higher in Ni-IDCM) [35]. One study did not find changes in expression during viral myocarditis [36]. Although these results are not consistent, the possible correlation with coxsackie infection increases its possible clinical application.

Two related miRNAs—miR-145a and miR-145b—may be associated with the differentiation of Th17 cells and TNF-alpha release [37]. Four studies have reported an overexpression of miR-145b [14,38,39,40], and one of miR-145a, during MCI [13]. In one study, both miRNAs showed a non-significant increase during the acute phase of MCI [22]. Other studies did not confirm this correlation. If only this evidence was available, these miRNAs would be qualified as the best LB candidates. However, these miRNAs were measured in 10 studies (miR-145b) and 3 studies (miR-145a). For the former miRNA, 7 out of 10 studies were performed on MCI cohorts and 3 on InfDCM cohorts. For the latter miRNA, 2 out of 3 studies were on MCI patients and 1 on patients with rheumatic carditis. No other study reported an overexpression of miR-145a or miR-145b in myocardial diseases. Thus, we decided to underline their possible role and the need for further investigation.

### 4.3. Possible Roles of cfDNA

None of the included studies validated the usability of cfDNA. However, this technique may provide novel possibilities for LB in MCI/InfDCM. This technique showed the ability to replace EMB in post-transplant patients using analysis of donor-derived cfDNA [41]. It was also proved that the analysis of methylation patterns in cfDNA is a sensitive and specific method for cardiomyocyte damage assessment [42]. On the other hand, we currently do not have any evidence that it could provide more information on inflammatory myocardial diseases than classical myocardial damage biomarkers (troponin and CK-MB).

### 4.4. Combining miRNAs

The accuracy of liquid biopsy diagnostics can be significantly improved by combining results of several measurements including different miRNA levels, as well as of commonly available clinical parameters (i.e., HR, RR, WBC, CRP, troponin, CK-MB, and BMI). Computational methods, such as principal component analysis, allow the best parameters for differentiation in patients’ diagnosis to be found.

The above analysis was implemented by Calderon-Domingeuz et al. Using multivariate logistic regression, they identified seven predictors of severe idiopathic DCM (presence of left bundle branch block, left-ventricular end-systolic diameter, systolic blood pressure, smoking habits, and the concentrations of miR-130b, miR-150, and miR-210). This model allowed them to increase the AUC for distinguishing severe DCM from moderate DCM from 0.78 (for the best miRNA—miR-145) to 0.96. However, the miRNA yielding the highest AUC on its own was not identified as a prediction factor in this model [43].

### 4.5. Recommendations for Future Studies

Serum miRNAs may become a marker of myocardial inflammation in the future. On the other hand, the high variability in the results between the reviewed studies puts their applicability into question. More attention should be paid to the design, methodology, and reporting in future studies. In addition, an appropriate selection of patients needs to be considered. In accordance with the ESC guidelines, if autoimmune and/or infectious MCI or DCM is suspected, EMB should be performed for a definite diagnosis. Furthermore, biopsy samples should be assessed by a comprehensive panel of histological, immunohistochemical, and virological assays [2]. Genetic testing should additionally be considered in children with DCM. The clinical diagnosis of “idiopathic dilated cardiomyopathy” seems to be overused in many studies. In our opinion, this term should be applied only in cases where available diagnostic methods cannot differentiate the disease etiology.

This study revealed that the two-step study design (screening by a high-throughput method and validation by qPCR) provided better and more consistent results. In the authors’ opinion, the use of dPCR instead of qPCR should be considered in future studies due to increased precision, reproducibility, and sensitivity [44]. Researchers should use appropriate miRNA reference genes—housekeeping genes (gene stably expressed in tissues, regardless of disease) and internal controls (synthetic miRNA for the assessment of reaction efficiency) for standardization of the results. Further studies are necessary to determine which housekeeping genes are the most optimal for assessing miRNA in cardiac samples. For qPCR, MIQE guidelines ought to be followed [8]. Experiments reporting diagnostics methods should follow STARD 2015 guidelines [45]. It would also be beneficial to provide detailed information about subjects and the results of the EMB assessment, as well as complete results of high-throughput methods.

Animal studies provide an opportunity to model specific disease conditions, i.e., the early phase of viral myocarditis or autoimmune myocarditis. Their results can provide increased repeatability and more samples at different time points. This leads to a better understanding of the specific conditions and dynamics of each miRNA release. Such results can then be translated into clinical data, as in one of the included studies [9]. However, the expression of some miRNAs can sometimes vary between different species [46]. For this reason, all miRNA markers discovered in animals should be confirmed in humans.

Future studies should also study the application of relevant techniques for creating multicomponent factors consisting of different parameters that can improve diagnostic accuracy.

### 4.6. Study Limitations

Although as much attention as possible was paid to providing accurate and objective results, this study has some limitations. Thus, the authors prepared a screening scale. This screening scale was based on positive criteria only (it did not regard results concerning non-specificity or non-repeatability in other studies). The final assessment was based on databases, available sources, and the authors’ experience, which may lead to bias. Applying statistical methods and performing metanalysis may provide more accurate results. However, it is not certain that the published data can be compared in this way.

Due to the nature of this study, we could not completely fulfill the PRISMA guidelines [7]. In this review, the PRISMA criteria for risk of bias assessment, statistical synthesis methods, and certainty assessment were not applied. In addition, the review protocol has not been registered.

In the authors’ opinion, future studies should not solely focus on the miRNAs identified in this study, but also on high-throughput techniques, such as miRNA sequencing whenever possible. Based on the authors’ experience, the cost of these techniques is comparable to that of several qPCR assays.

## 5. Conclusions

**Figure 3:**
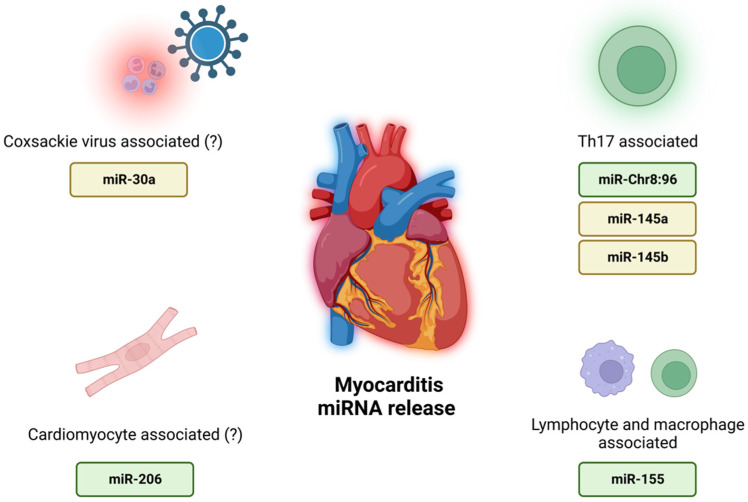
Sources of the proposed miRNAs; biomarker candidates are shown in green boxes, and potential biomarker candidates are shown in yellow boxes. Created with BioRender.com. Accessed on 7 September 2022.

To the best of authors’ knowledge, this is the first systematic review of liquid biopsy markers as potential candidates for the detection of inflammatory myocardial diseases. In the light of current studies, three miRNAs: (1) miR-Chr8:96, (2) miR-155, and (3) miR-206 are the best candidates for potential LB markers of myocardial inflammatory state. Serum levels of the above-mentioned miRNAs positively correlate with the diagnosis of MCI/InfDCM and show high specificity. Further studies are needed to provide additional evidence for the usefulness of these miRNAs in inflammatory myocardial pathology. Results of this systematic review are summarized in Figure 3. 

## Figures and Tables

**Table 1:** Inclusion criteria.

The article describes a clinical study with measurements of cfDNA level in plasma/serum or miRNA in plasma/serum/myocardial tissue. All types of blood derivatives (peripheral blood mononuclear cells (PBMC), CD4^+^ cells, or exosomes) were also considered eligible.
The article involves a group of patients with myocarditis, inflammatory dilated cardiomyopathy, or non-ischemic idiopathic dilated cardiomyopathy (criteria of disease classification are described below).
The article involves a control group (positive i.e., healthy subjects, or negative i.e., patients with myocardial infarction, ischemic dilated cardiomyopathy, or other myocardial disease).

**Table 2:** Exclusion criteria.

The article is a case study or a review.
The article describes only an in vitro or animal study.
The article does not involve an independent control group.
The study group suffers from other serious cardiac diseases (i.e., amyloidosis or infarction).
The article is retracted.
The article presents only data not useful in the context of this study, for example, there is no possibility of extracting information regarding particular miRNAs.

**Table 3:** Study groups classification criteria.

Disease	Criteria
Myocarditis (MCI)	The study group was classified into this group when the following clinical diagnosis was met: myocarditis, acute myocarditis, fulminant myocarditis, viral myocarditis, autoimmune myocarditis, or rheumatic carditis. Chagas myocarditis was considered inappropriate. There was no need to provide additional evidence for this diagnosis.
Inflammatory Dilated Cardiomyopathy (InfDCM)	The study group was classified into this group when patients met the diagnostic criteria of DCM, ischemic heart disease was excluded, and inflammation was proven with myocardial biopsy.
Non-ischemic Idiopathic Dilated Cardiomyopathy (Ni-IDCM)	The study group was classified into this group when patients met the diagnostic criteria of DCM and ischemic heart disease were excluded. The cause of the disease must have been undetermined.

**Table 4:** miRNA screening scale.

Utility as LB Marker	Criteria
Very high (5)	Micro RNA was selected in at least one study using a screening method (microarray or miRNAseq) *. Micro RNA was assessed in at least two independent studies or one study involving at least two independent groups. Studies compared miRNA levels in MCI/InfDCM, a healthy control group, and at least two other myocardial diseases. Studies provide valid evidence for patient group homogeneity. At least one study provided a ROC curve for distinguishing MCI/InfDCM from healthy patients or patients with other diseases; AUC > 0.9. If studies compared miRNA expression in other diseases, miRNA must have shown a potential for MCI/InfDCM specificity.
High (4)	Micro RNA was selected in at least one study based on a screening method (microarray or miRNAseq) *. Studies compared miRNA levels in MCI/InfDCM, a healthy control group, and at least one myocardial disease. At least one study provided a ROC curve for distinguishing MCI/InfDCM from healthy patients or patients with other diseases; AUC > 0.7/at least one study provides strong evidence for miRNA specificity and sensitivity. If studies compared miRNA expression in other diseases, miRNA must have shown a potential for MCI/InfDCM specificity.
Medium (3)	Studies compared miRNA levels in MCI/InfDCM, a healthy control group, and at least one myocardial disease. At least one study provides a statistically significant correlation between miRNA concentration and MCI/InfDCM occurrence. If studies compared expression in MCI/InfDCM with other diseases, miRNA must have shown a potential for MCI/InfDCM specificity.
Low (2)	Studies compared miRNA levels in MCI/InfDCM and a healthy control group. At least one study provides a statistically significant correlation between miRNA concentration and MCI/InfDCM occurrence. If studies compared expression in MCI/InfDCM with other diseases, miRNA must have shown a potential for MCI/InfDCM specificity.
Very low (1)	Studies did not include evidence for the correlation of miRNA with MCI/InfDCM or miRNA was non-specific.

* These criteria applies to two-step studies, where miRNAs were selected by high-throughput methods (i.e., miRNA sequencing or microarrays), and then assessed using a low-throughput method (qRT-PCR). This allows the selection of the most up- or downregulated miRNAs in a study group.

**Table 5:** Results of screening.

Utility as LB Marker	miRNAs
Very high (5)	let-7f, miR-1, miR-27b, miR-142, miR-143, miR-155, miR-223, miR-Chr8:96
High (4)	miR-29b, miR-30a, miR-106a, miR-125b, miR-133a, miR-133b, miR-146a, miR-146b, miR-181b, miR-192, miR-197, miR-206, miR-320a, miR-4763, mmu-miR-93, mmu-miR-379
Medium (3)	miR-15b, miR-16, miR-17, miR-21, miR-27a, miR-125a, miR-151a, miR-185, miR-194, miR-205, miR-208, miR-208a, miR-212, miR-220c, miR-222, miR-342, miR-489, miR-499, miR-660, miR-671
Low (2)	let-7g, miR-7, miR-10b, miR-26a, miR-26b, miR-29, miR-92a, miR-92b, miR-93, miR-98, miR-99b, miR-107, miR-130b, miR-133, miR-135b, miR-147, miR-148a, miR-150, miR-181d, miR-190a, miR-199b, miR-217, miR-221, miR-297, miR-301a, miR-301b, miR-302a, miR-338, miR-339, miR-361, miR-362, miR-363, miR-365a, mir-378, miR-381, miR-422a, miR-423, miR-451-DICER1, miR-451a, miR-454, miR-455, miR-486, miR-495, miR-496, miR-511, miR-518f, miR-520e, miR-543, miR-544, miR-551b, miR-590, miR-595, miR-601, miR-618, miR-770, miR-875, miR-889, miR-1180, miR-1261, miR-1290, miR-3064, miR-3135b, miR-3148, miR-3908, miR-4701, miR-4793, miR-5571, miR-6785, miR-6796, miR-6807, miR-6847, miR-6849, miR-6856, miR-7844
Very low (1)	let-7a, let-7b, let-7c, let-7i, miR-9, miR-10a, miR-16-2, miR-19a, miR-19b, miR-20a, miR-20b, miR-23a, miR-23b, miR-24, miR-24-1, miR-25, miR-28, miR-29a, miR-30b, miR-30c, miR-30e, miR-34a, miR-99a, miR-100, miR-101, miR-103a, miR-122, miR-125, miR-126, miR-132, miR-134, miR-139, miR-140, miR-141, miR-144, miR-145, miR-154, miR-191, miR-193a, miR-195, miR-196a, miR-199a, miR-200c, miR-208b, miR-210, miR-214, miR-215, miR-218, miR-296, miR-323, miR-324, miR-326, miR-365, miR-375, miR-378a, miR-423-5P, miR-449, miR-483, miR-497, miR-499a, miR-502, miR-532, miR-624, miR-629, miR-3940, miR-3960, miR-4821, miR-5010, miR-5088

Green-labeled miRNAs are finally assessed as MCI/InfDCM LB biomarker candidates. Yellow-labeled miRNAs are finally assessed as MCI/InfDCM LB biomarker potential candidates.

## Data Availability

All data presented in this study are available in the article and Supplementary Materials.

## Supplementary Materials

The following supporting information can be downloaded at: https://www.mdpi.com/article/10.3390/biom12101476/s1, Supplementary Materials S1: Search keys utilized in database searches; Tables S1 and S2: A complete list of excluded studies with exclusion reasons; Table S3: All data included in studies; Table S4: Descriptions of reasons for exclusion of assessed miRNA. The references of papers cited in the Supplementary Materials are also cited here for the sake of completedness [47,48,49,50,51,52,53,54,55,56,57,58,59,60,61,62,63,64,65,66,67,68,69,70,71,72,73,74,75,76,77,78,79,80].
