# Supplementary material for: A Systematic Review of miRNA and cfDNA as Potential Biomarkers for Liquid Biopsy in Myocarditis and Inflammatory Dilated Cardiomyopathy
Source: Biomolecules. 2022 Oct 13;12(10):1476. doi: 10.3390/biom12101476 (PMC9599237; doi:10.3390/biom12101476)
Supplement: Supplementary file 1 [file biomolecules-12-01476-s001.zip › biomolecules-1931636-supplementary/Supplementary materials/Supplementary material S1.pdf]

Supplementary Material S1

Search keys utilized in database searches

PubMed

(myocarditis[Title] OR (dilated[Title] AND cardiomyopathy[Title]) OR dcm[Title]) AND (cfdna[Title] OR (circulating[Title] AND free[Title] AND dna[Title]) OR (cell[Title] AND free[Title] AND dna[Title]) OR mirna[Title] OR microrna[Title] OR (micro[Title] AND rna[Title]))

Scopus

(myocarditis[Title] OR (dilated[Title] AND cardiomyopathy[Title]) OR dcm[Title]) AND (cfdna[Title] OR (circulating[Title] AND free[Title] AND dna[Title]) OR (cell[Title] AND free[Title] AND dna[Title]) OR mirna[Title] OR microrna[Title] OR (micro[Title] AND rna[Title]))

Web of science

(myocarditis[Title] OR (dilated[Title] AND cardiomyopathy[Title]) OR dcm[Title]) AND (cfdna[Title] OR (circulating[Title] AND free[Title] AND dna[Title]) OR (cell[Title] AND free[Title] AND dna[Title]) OR mirna[Title] OR microrna[Title] OR (micro[Title] AND rna[Title]))

Embase

(myocarditis[Title] OR (dilated[Title] AND cardiomyopathy[Title]) OR dcm[Title]) AND (cfdna[Title] OR (circulating[Title] AND free[Title] AND dna[Title]) OR (cell[Title] AND free[Title] AND dna[Title]) OR mirna[Title] OR microrna[Title] OR (micro[Title] AND rna[Title]))
