# Supplementary material for: A Systematic Review of miRNA and cfDNA as Potential Biomarkers for Liquid Biopsy in Myocarditis and Inflammatory Dilated Cardiomyopathy
Source: Biomolecules. 2022 Oct 13;12(10):1476. doi: 10.3390/biom12101476 (PMC9599237; doi:10.3390/biom12101476)
Supplement: Supplementary file 1 [file biomolecules-12-01476-s001.zip › biomolecules-1931636-supplementary/Supplementary materials/Table S3.pdf]

Study	ID	Assessed microRNA (in validation study if applicable)	Number of patients (study group / control)	Patient age	% of female patients	Type of sample	Method of miRNA screening	Method of miRNA isolation	Method of measurement	Reference gene	Clinical diagnosis of study group	Clinical diagnosis of control group / groups
[1]	1	miR-Chr8:96,<br>miR-483-5p,<br>miR-21-5p,<br>miR-212-3p,<br>miR-132-3p	Main cohort: 42/170<br>Validation cohort I: 34/11<br>Validation cohort II: 35/20<br>Validation cohort III: 40/49<br>Validation cohort IV: 0/152	**In main cohort:**<br>Healthy control: 42,31±1,21<br>Acute myocarditis: 40,36±19,40<br>STEMI: 60,31±12,61<br>NSTEMI: 65,69±14,14	**In main cohort:**<br>Healthy control: 48,75<br>Acute myocarditis: 40,36<br>STEMI: 60,31<br>NSTEMI: 65,69	Serum	Murine experiment and microRNA analysis (microarrays and qPCR)	Invitrogen miRVana miRNA Isolation kit	qRT-PCR,<br>Exiqon miRCURY LNA Universal RT microRNA PCR kit, Exiqon microRNA LNA PCR primer sets, Applied Biosystem SYBR green PCR mix, for miR-Chr8:96 own primers	UniSp5 or UniSp2 (Exiqon,<br>RNA Spike-in kit),<br>miR-423-3p and miR-103a-3p	MCI	Healthy subjects, STEMI, NSTEMI, acute coronary syndrome MINOCA, myocardial infraction
[2]	22	miR-16-5p<br>miR-21-5p<br>miR-29a-5p<br>miR-133a-3p<br>miR-191-5p<br>miR-320a<br>miR-423-5p	DCM:53<br>IHD:34<br>Control:48	DCM: 59±12<br>IHD: 66±9<br>Control 64±5	DCM:32<br>IHD:12<br>Control: 62	Plasma	NA	Qiagen miRNEasy kit	qRT-PCR,<br>Exiqon miRCURY LNA™ Universal RT microRNA PCR, Polyadenylation, and cDNA synthesis kit	UniSp6 spike-in	Ni-IDCM	Healthy Controls, Ischemic Heart Disease
[3]	26	let-7a-5p<br>let-7g-5p<br>miR-1-3p<br>miR-16-5p<br>miR-16-2-3p<br>miR-19b-3p<br>miR-25-3p<br>miR-29a-3p<br>miR-30b-5p<br>miR-30c-3p<br>MIR-130b-3p<br>miR-133a-3p<br>miR-133b-3p<br>miR-142-3p<br>miR-145-5p<br>miR-150-5p<br>miR-192-5p<br>miR-199a-3p<br>miR-210-3p<br>miR-215-3p<br>miR-324-3p<br>miR-363-3p<br>miR-454-3p<br>miR-532-5p<br>miR-629-5p<br>miR-660-5p	MODERATE<br>Idiopathic DCM: 40<br>Ischemic DCM: 46<br><br>SEVERE<br>Idiopathic DCM: 15<br>Ischemic DCM: 14<br><br>Control: 44	MODERATE<br>Idiopathic DCM: 67.6 ± 9.4<br>Ischemic DCM: 68.0 ± 8.3<br><br>SEVERE<br>Idiopathic DCM: 66.5 ± 8.4<br>Ischemic DCM: 68.7 ± 8.4<br><br>Control: 39.1 ± 12.8	MODERATE<br>Idiopathic DCM: 30<br>Ischemic DCM: 23,9<br><br>SEVERE<br>Idiopathic DCM: 40<br>Ischemic DCM: 7.1<br><br>Control: 47.7	Plasma	qRT-PCR Panel	Qiagen miRNeasy Serum/Plasma Kit	qRT-PCR, Qiagen miRCURY LNA RT Kit, miRCURY LNA SYBR Green PCR Kit, Qiagen 96-wells Pick-&-Mix microRNA PCR plates	cel-miR-39-3p, UniSp6	Ni-IDCM	Healthy Controls, Ischemic DCM
[4]	32	miR-1<br>miR-134<br>miR-423-5p<br>miR-489<br>miR-496<br>miR-5010<br>miR-5088	20/5	DCM: 57.4±15.3<br>Control: 53±6	DCM: 35<br>Control:40	Serum	miRNA analysis-microarray miRNA analysis previously hybridized 3D-genes human miR chips (Toray Industries, Inc., Tokyo Japan)	NA	qRT-PCR	miR-16 / miR-423-3p	Ni-IDCM	Healthy Controls
[5]	33	Let-7f-5p<br>Let-7g-5p<br>Let-7i-5p<br>miR-142-5p<br>miR-126-3p<br>miR-143-3p<br>miR-24-3p<br>miR-26a-5p<br>miR-27a-3p<br>miR-27b-3p<br>miR-98-5p	30/16	DCM: 4,15 ± 3.85<br>Control: 5,97 ± 4,41	DCM: 56,67<br>Control: 43,75	Serum	Illumina sequencing	mirVana miRNA (Ambion, Thermo Fisher Scientific Inc, Waltham, MA USA).	qRT-PCR	NA	Ni-IDCM	Healthy Controls
[6]	49	miR-3135b<br>miR-3908<br>miR-5571-5p	20/19	Control: 56,7 ± 9,1<br>DCM: 59,2 ± 13,1	Control: 20,0<br>DCM: 15,8	Serum	Exiqon miRCURYTM LNA Array	Invitrogen TRIzol, Qiagen Rneasy Mini Kit	qRT-PCR	miR-191-5p	Ni-IDCM	Healthy Controls
[7]	70	miR-29b<br>miR-125b<br>miR-220c-3p	FM:10<br>MI:10<br>Control: 7	FM: 37,8 ± 4,32<br>MI: 36,09 ± 5,08<br>Control: 35,23 ± 5,12	FM: 30<br>MI:40<br>Control: 42,9	Plasma	miRNA Array	King Biotech Rnasy Mini Kit	qRT-PCR	NA	MCI	Healthy Controls, Myocardial infarction

		miR-142<br>miR-200c-3p										
[8]	76	let-7f<br>miR-93<br>miR-197<br>miR-21<br>miR-30a-5p<br>miR-223<br>miR-379<br>mmu-miR-379	Control: 85<br>iDCM: 343<br>DCM: 71	Control: 44.5 ± 16.5iDCM: 49 ± 29<br>DCM: 55 ± 11	Control:69<br>iDCM: 22<br>DCM: 47	Serum	TaqMan Open Array (Human MicroRNA Panels A and B)	Invitrogen mirVANA™ PARIS™ RNA and Native Protein Purification Kit	qRT-PCR, Thermo Fisher Megaplex stem-loop RT primer, TaqMan MicroRNA Reverse Transcription Kit	miR-30a-3p	InfDCM	Healthy Controls, Non-inflammatory DCM
[9]	82	miR-99b<br>miR-147<br>miR-155<br>miR-194-5p<br>miR-205<br>miR-218<br>miR-302a<br>miR-454<br>miR-518f<br>miR-544<br>miR-618<br>miR-875-3p	23/26	Control: 52.23 ± 40<br>DCM: 48.78 ± 43<br>**months old**	Control 46,2<br>DCM: 43,5	Plasma	NA	Qiagen miRNeasy Mini Kit	qRT-PCR, Life Technologies TaqMan miRNA Reverse Transcription Kit, MegaPlex Human RT Primer Pool A, TaqMan PreAmp Master Mix, MegaPlex Human Preamplification Primers, TaqMan Universal PCR Master Mix, Fluidigm Dynamic Array 96.96 chips	miR-483-5p	Ni-IDCM	Healthy Controls
[10]	86	miR-21<br>miR-155<br>miR-181b	Control: 31<br>Ischemic DCM: 31<br>Idiopathic DCM: 41	Control: 50,29 ± 7,62<br>Ischemic DCM: 49,31 ± 8,99<br>Idiopathic DCM: 55,26±7,47	Control: 56,8<br>Ischemic DCM: 32,3<br>Idiopathic DCM: 34,1	Serum	NA	Ambion TRIzol LS	qRT-PCR, Promega Cat. #A3500, primers sequences provided	U6	Ni-IDCM	Healthy Controls, Ischemic Cardiomyopathy
[11]	96	miR-1<br>miR-122<br>miR-133a<br>miR-208b<br>miR-223<br>miR-449<br>miR-29b<br>miR-146a<br>miR-146b<br>miR-21<br>miR-126<br>miR-155	Control: 20<br>AMI: 36<br>MCI:14 (acute phase) / 20 (post VM)<br>DD: 59<br>AHF: 33	Control: 32,1 ± 7,3<br>AMI: 62 ± 13<br>MCI: 32,7 ± 16,3 (acute phase) / 37,3 ± 19,4 (post VM)<br>DD: 69 ± 8<br>AHF: 76,3 ± 8,4	Control: 40<br>AMI: 33,3<br>MCI: 14,2 (acute phase) / 15 (post VM)<br>DD: 44<br>AHF: 60,1	Plasma	NA	Ambion mirVana PARIS kit	qRT-PCR, Qiagen miScript kit, Quanta Biosciences BR SYBR-green supermix for IQ, Qiagen MiScript primers sets	Mix of 3 spiked-in synthetic miRNA	MCI	Healthy Controls, Acute Myocardial Infarction, Diastolic Dysfunction, Acute Heart Failure
[12]	111	let-7b-5p<br>let-7c-5p<br>miR-1-3p<br>miR-15b-5p<br>miR-17-5p<br>miR-19a-3p<br>miR-19b-3p<br>miR-20a-5p<br>miR-20b-5p<br>miR-23a-3p<br>miR-24-3p<br>miR-27a-3p<br>miR-28-5p<br>miR-30e-5p<br>miR-99b-5p<br>miR-100-5p<br>miR-101-3p<br>miR-103a-3p<br>miR-106a-5p<br>miR-125b-5p<br>miR-126-3p<br>miR-126-5p<br>miR-140-5p<br>miR-191-5p<br>miR-195-5p<br>miR-199a-3p<br>miR-214-3p<br>miR-222-3p<br>miR-342-3p<br>miR-378a-3p	Control: 10<br>Ischemic DCM: 25<br>Idiopathic DCM: 25	Control: 59,8 ± 7,8<br>Ischemic DCM: 59,8 ± 9,4<br>Idiopathic DCM: 58,7 ± 12,7	Control: 40<br>Ischemic DCM: 12<br>Idiopathic DCM: 32	Plasma	NA	Qiagen miScript RT II Kit	qRT-PCR, Qiagen QuantiTect SYBR Green PCR Master Mix, miScript Primer Assay	SNORD61, miRTC	Ni-IDCM	Healthy Controls, Ischemic DCM
[13]	122	miR-381	26/33	NA	NA	Serum	NA	Yeasen TRIzol	qRT-PCR, Tiangen miRcute miRNA cDNA First Strand	U6	MCI	Healthy Controls

									Synthesis Kit, Tiangen miRNA RT-PCR kit, own primers (sequences provided)			
[14]	125	miR-25	Control: 23<br>HHD: 15<br>HHDF: 12<br>DCM: 9<br>OMI: 8	Control: 62±3<br>HHD: 61.4±2.4<br>HHDF: 70.6±2.6<br>DCM: 70.6±2.6<br>OMI: 72.8±3.6	Control: 34,8<br>HHD: 61,1<br>HHDF: 44,4<br>DCM: 25<br>OMI: 37,5	Serum	NA	Invitrogen TRIzol LS	qRT-PCR, Promega MMLV Reverse Transcriptase Kit, Roche Fast Start Universal SYBR Green Master Mix, Ribobio miRNA primers	Ribobio ath-miR156a	Ni-IDCM	Healthy Controls, Hypertensive Heart Disease, Hypertensive Heart Disease leading resulting in Heart Failure, Remote Infarct
[15]	151	miR-21<br>miR-126<br>miR-133<br>miR-146b<br>miR-155<br>miR-206	InfDCM: 60<br>DCM: 29	InfDCM: 47 ± 15<br>DCM: 43 ± 14	InfDCM: 28<br>DCM: 24	Plasma	NA	miRNeasyMini Kit	qRT-PCR, miScript PCR system	miR-39–1	InfDCM	Non-inflammatory DCM
[16]	158	miR-21<br>miR-26<br>miR-29<br>miR-30<br>miR-133a	70 / 7	NA	NA	EMB	NA	Life Technologies mirVana kit	qRT-PCR, TaqMan Advanced MicroRNA cDNA Synthesis Kit, TaqMan Advanced Master Mix, TaqMan Advanced Assays	NA	Ni-IDCM	Healthy Controls
[17]	160	miR-1-3p<br>miR-21-5p	40/29	24 ± 7 / 23 ± 6	80 / 79	Peripheral blood mononuclear cells	NA	Stem Cell Technologies Lymphoprep / Ambion Tri-Reagent	qRT-PCR, Clontech miR-X miRNA First-Strand Synthesis Kit, Kapa Biosystems KAPA SYBR FAST qPCR Kit	U6	MCI	Healthy Controls
[18]	171	miR-126<br>miR-146a<br>miR-155<br>miR-361-5p<br>miR-423-5p	45/39	47.76 ± 12.28 / 47.59 ± 11.85	28.9 / 35.9	Plasma	NA	mirVana PARIS kit (AM1556, Ambion)	qRT-PCR, Applied Biosystems TaqMan MicroRNA Reverse Kit, TaqMan! MicroRNA Assay, Universal PCR Master Mix	Qiagen cel-miRNA-39	Ni-IDCM	Healthy Controls
[19]	172	miR-185	50 / 41	45 ± 0.28 / 42 ± 0.31	38 / 39	B cells isolated from PBMC, and plasma	NA	TRIzol reagent (Invitrogen)	qRT-PCR, TaqMan MicroRNA Reverse Transcription kit, TaqMan Universal PCR Master Mix Kit	NA	Ni-IDCM	Healthy Controls
[20]	202	miR-217<br>miR-543	30 / NA	MCI: from 9 months to 12 years old	MCI: 43	Blood	NA	TRIzol® reagent (Invitrogen; Thermo Fisher Scientific, Inc.)	qRT-PCR, PrimeScript RT Reagent Kit (Takara Bio, Inc.), SYBR Premix Ex Taq (Takara Bio, Inc.), house made primers (sequence provided)	U6	MCI	Healthy subjects
[21]	203	miR-21<br>miR-30a<br>miR-125b<br>miR-146a<br>miR-155<br>miR-192<br>miR-223-3p<br>miR-320a	FM: 99<br>Non-FM: 32<br>Control: 105	FM: Median:40<br>NFM: Median 33	FM: 40<br>NFM 28.1	Serum exosomes	Illumina sequencing (Illumina, USA)	mirVana miRNA isolation kit (Thermo Fisher, MA, USA)	qRT-PCR, Ribobio miRNA qPCR primer set	miR-39	MCI	Healthy subjects
[22]	209	miR-98	50 / 50	NA	NA	Plasma	NA	RNA extraction kit (Invitrogen, Chicago, IL, USA)	qRT-PCR, Toyobo SYBR Green	U6	MCI	Healthy subjects
[23]	210	miR-1<br>miR-10a-5p<br>miR-16-5p<br>miR-21-5p<br>miR-24-3p<br>miR-92a-3p<br>miR-125<br>miR-130b-3p<br>miR-132-3p<br>miR-146a-5p	36 / 35	12,1 ± 2,1 / 11,1 ± 2,3	52,8 / 45.7	Plasma	NA	Roche High Pure miRNA Isolation Kit	qRT-PCR, Applied Biosystems TaqMan MicroRNA Reverse Transcription Kit, Applied Biosystems TaqMan PreAmp Master Mix 29, Applied Biosystems Megaplex Human Primer Pools Set v3.0, Fluidigm	NA	MCI	Healthy subjects

		miR-155-5p<br>miR-196a-5p<br>miR-221-3p<br>miR-223-3p<br>miR-320a<br>miR-499a-5p							96.96 Dynamic Array Integrated Fluid Circuits			
[24]	231	miR-93<br>miR-106a<br>miR-146b<br>miR-155<br>miR-511<br>miR-212<br>miR-889	4 / 6	39 ± 20 / 38 ± 11	0 / 33.3	EMB	NA	Ambion miRVANA isolation kit	MicroArray (Illumina Human miRNAv2 Expression Panel arrays)	NA	MCI	Healthy subjects
[25]	243	miR-29	52 / 32	Adult population<br>51 / 42,5<br><br>Pediatric population<br>4 / 12,7	Adult population<br>50 / 50<br><br>Pediatric population<br>52 / 46	EMB	NA	Ambion MirVanaTM RNA Isolation Kit	qRT-PCR, Qiagen miScript Reverse Transcription Kit, Primers sequences avaiable	U6	Ni-IDCM	Healthy subjects
[26]	251	miR-151a-3p<br>miR-3960<br>miR-4763-3p<br>miR-4821	20 / 10	36,55 ± 18,64 / 28,5 ± 1,84	55/30	Plasma	Agilent miRNA Microarray System	Invitrogen TRIzol-LS, Qiagen RNeasy mini kit	qRT-PCR, Ribobio Bulge-Loop miRNA qRT-PCR Starter Kit, Power SYBR Green PCR Master Mix, Ribobio primers	Cel-miR-39-3p	MCI	Healthy subjects
[27]	284	miR-21<br>miR-29b<br>miR-34a<br>miR-99a<br>miR-107<br>miR-125b<br>miR-139<br>miR-142-3p<br>miR-154<br>miR-326<br>miR-497<br>miR-502	Ni-IDCM: 19<br>ICM:15<br>Controls: 23	Ni-IDCM: 63,8 ± 1,8<br>ICM: 67 ± 1,8<br>Controls: NA	Ni-IDCM: 31,6<br>ICM:13<br>Controls: NA	PBMC	qRT-PCR panel	NA	qRT-PCR, Appplied Biosystems TaqMan Real Time Assay	miR-16	Ni-IDCM	Healthy subjects, Ischemic Cardiomyopathy
[28]	287	miR-1<br>miR-21<br>miR-27b<br>miR-30a<br>miR-125a<br>miR-133a<br>miR146b<br>miR-148a<br>miR-155<br>miR-181d	23 / 12	NA	NA	Serum exosomes	NA	TRIZOL Kit (Invitrogen, United States)	qRT-PCR, TaqMan miRNA RT-Real Time PCR, TaqMan miR- NA RT-Real Time PCR, miRNA-specific TaqMan MGB probes	miR-16	MCI	Healthy subjects
[29]	305	miR-23b-3p<br>miR-24-1-3p<br>miR-27b-3p<br>miR-155-5p	ICM: 9<br>Ni-IDCM: 9<br>HOCM: 12<br>Control: 9	ICM: 53 ± 5<br>Ni-IDCM: 52 ± 4<br>HOCM: 51 ± 6<br>Control: 52 ± 7	0	Heart tissue	NA	Bioline Trisure	qRT-PCR, Applied Biosystems cDNA for TaqMan miRNA, Applied Biosystems TaqMan MicroRNA Reverse Transcription Kit, TaqMan miRNA assays	RNU48	Ni-IDCM	Healthy subjects, Hypertrophic Cardiomyopathy, Ischemic Cardiomyopathy
[30]	334	miR-24	DCM: 8<br>ICM: 5<br>Control: 4	DCM: 41,1 ± 5,3<br>ICM: 56,4 ± 4,9<br>Control: 35,0 ± 4,6	NA	Heart tissue	NA	TRIZOL Kit (Invitrogen, United States)	qRT-PCR, Ribobio microRNA-specific reverse-transcription primers, Stratagen Brilliant II SYBR Green QPCR master mix	U6	Ni-IDCM	Healthy subjects, Ischemic Cardiomyopathy
[31]	352	miR-92b-5p<br>miR-192-5p<br>miR-320a	43 / 34	62±2 / 57±2	53.5 / 32.4	Serum exosomes	NA	miRNA Quick Extraction Kit (Bioteke, Beijing, China)	qRT-PCR	miR-451b	Ni-IDCM	Healthy subjects
[32]	380	miR-1<br>miR-21<br>miR-126<br>miR-133a<br>miR-146b<br>miR-155	76 / 22	45 ± 15 / 53 ± 13	19 / 45	EMB	NA	miRNeasy Mini Kit (Qiagen, Hilden, Germany)	qRT-PCR, Qiagen miScript PCR System, Qiagen Primers	NA	InfDCM	Ni-IDCM

		miR-206<br>miR-208b										
[33]	386	miR-1<br>miR-15b<br>miR-19a<br>miR-19b<br>miR-99b<br>miR-101<br>miR-125b<br>miR-133<br>miR-208<br>miR-214	ICM: 19<br>DCM: 25<br>AS: 13<br>Control:10	ICM: 6.6 ± 0.6<br>DCM: 6.0 ± 1.5<br>AS: 8.6 ± 0.7<br>Control: 5.8 ± 1.4	ICM: 11<br>DCM: 32<br>AS: 54<br>Control: 40	Heart tissue	Bead based method	TRIzol (Invitrogen, Carlsbad, CA)	qRT-PCR	NA	Ni-IDCM	Healthy subjects, Ischemic Cardiomyopathy, Aortic Stenosis
[34]	388	miR-21<br>miR-125b<br>miR-146b<br>miR-155<br>miR-208b<br>miR-214<br>miR-375<br>miR-499	8 / 8	NA	NA	EMB	NA	TRIzol (Invitrogen Life Technologies, Carlsbad, CA, USA)	qRT-PCR, Applied Biosystems TaqMan miRNA RT-Real Time PCR	U6	MCI	Healthy subjects
[35]	420	miR-199a<br>miR-199b<br>miR-214	42 / 20	45,4 ± 2 / 44,4 ± 3,9	9,5 / 35	Heart tissue	NA	Trizol reagent (Invitrogen)	qRT-PCR, Applied Biosystems TaqMan miRNA assays, Applied Biosystems TaqMan MicroRNA Reverse Transcription Kit	U6	Ni-IDCM	Healthy subjects
[36]	446	miR-1<br>miR-146b	119 / 110	6.7±2.9 / 7.2 ± 3.0	46.2 / 46	Serum	NA	TRIZOL Kit (Invitrogen, United States)	qRT-PCR, Applied Biosystems TaqMan MicroRNA Reverse Transcription Kit	Beta-actin	MCI	Healthy subjects
[37]	448	miR-1<br>miR-21<br>miR-27b<br>miR-29b<br>miR-30a<br>miR-125a<br>miR-133a<br>miR-146b<br>miR-148a<br>miR-155<br>miR-199a<br>miR-208b<br>miR-223<br>miR-375<br>miR-499	6 / 6	NA	NA	Heart tissue	NA	Trizol (Invitrogen, Carlsbad, CA, USA)	qRT-PCR, Applied Biosystems aqMan MicroRNA Reverse Transcription Kit, TaqMan Universal PCR Master Mix, iRNA-specific TaqMan MGB probes	U6	MCI	Healthy subjects
[38]	463	miR-1<br>miR-7<br>miR-29b<br>miR-125b<br>miR-145<br>miR-181b<br>miR-214<br>miR-342<br>miR-378	50 / 20	54 ± 1 / 51 ± 2	34 / 55	Heart tissue	miRNA microarray (Ohio State Comprehensive Cancer Centre, version 3.0)	TRizol (Invitrogen)	qRT-PCR, Taqman real time mix	RNU6B	MCI	Healthy subjects
[39]	669	miR-1-3p<br>miR-17-5p<br>miR-30c-5p<br>miR-107<br>miR-133a-5p<br>miR-193a-5p<br>miR-208a-5p<br>miR-301a-3p<br>miR-301b-3b<br>miR-495-3p<br>miR-1261	Pediatric<br>36 / 13<br><br>Adult:<br>11 / 13	NA	Pediatric<br>54 / 53<br><br>Adult<br>3 / 39	Heart tissue	Illumina sequencing	Qiagen QIAzol / Ambion mirVana Kit	qRT-PCR, miScript SYBR Green PCR Kit, Qiagen miScript II cDNA synthesis, miRNAs primers provided	18s	Ni-IDCM	Healthy subjects

[40]	679	miR-146b	48 / 40	6,46 ± 2,17 / 6,37 ± 2,26	47.92 / 52.5	Aerum	NA	total miRNAs extraction kit (Qiagen, Germany, HZ101-633)	qRT-PCR, M-MLV reverse transcription kit (solarbio, USA, RP1100); qReal-time PRC kit (Invitrogen, Grand Island, NY, USA, article number: C28025-032); SYBR Green qPCR Master Mix Kit (Medchemexpress, USA, HY-K0501 Ltd.)	U6	MCI	Healthy subjects
[41]	731	Let-7f-5p<br>Let-7g-5p<br>Let-7i-5p<br>miR-24-3p<br>miR-26a-5p<br>miR-27a-3p<br>miR-27b-3p<br>miR-98-5p<br>miR-126-3p<br>miR-142-5p<br>miR-143-3p	16 / 12	5.27±4.13 / 5.49±3.69	62.5 / 67	Serum	Illumina sequencing	NA	qRT-PCR	NA	Ni-IDCM	Healthy subjects
[42]	943	miR-206<br>miR-297<br>miR-451a<br>miR-486-3p<br>miR-595<br>miR-1180-3p<br>miR-3064-5p<br>miR-3148<br>miR-4701-3p<br>miR-4793-3p<br>miR-6785-5p<br>miR-6796-5p<br>miR-6807-5p<br>miR-6847-5p<br>miR-6849-5p<br>miR-6856-5p<br>miR-7844-5p	45 / 45	50 ± 14 / 52 ± 12	33.3 / 37.8	CD4+ T cells	Microarray Affymetrix GeneChip® miRNA 4.1 arrays	TRIZOL Kit (Invitrogen, United States)	microArrray GeneChip® miRNA 4.1 arrays (Affymetrix)	U6	Ni-IDCM	Healthy subjects
[43]	1033	miR-208b	DCM:5<br>ICM:5<br>Control:5	DCM: 39 ± 10<br>ICM: 57 ± 11<br>Control: 29 ± 9	DCM:60<br>ICM:20<br>Control:40	Heart tissue	NA	Guanidinium thiocyanate-phenol-chloroform method	qRT-PCR, Exiqon miRCURY LNA™ Universal RT microRNA PCR kit	miR-103a-3p	Ni-IDCM	Healthy subjects, Ischemic Cardiomyopathy
[44]	1304	miR-9-3p<br>miR-16-2-3p<br>miR-21-3p<br>miR-21-5p<br>miR-144-3p<br>miR-144-5p<br>miR-338-3p<br>miR-363-3p<br>miR-451a<br>miR-551b-3p<br>miR-770-5p	NA	NA	NA	Heart tissue	miRNA microarray, Affymetrix 4.0 (OE Biotech's, Shanghai, China)	TRIzol; RNeasy mini kit (Qiagen, Hilden, Germany)	qRT-PCR	NA	Ni-IDCM	Healthy subjects
[45]	1348	miR-21<br>miR-126<br>miR-133a<br>miR-146b<br>miR-155<br>miR-206	81 / 22	NA	NA	Heart tissue	NA	NA	qRT-PCR	NA	MCI	Non-inflammatory DCM
[46]	1442	miR-208	56 / 21	NA	NA	Heart tissue	NA	Ambion mirVana Paris miRNA isolation kit	qRT-PCR, high-capacity cDNA Archive Kit, TaqMan microRNA assays	U6	Ni-IDCM	Healthy subjects
[47]	1454	miR-1<br>miR-21<br>miR-26b<br>miR-133a<br>miR-208b<br>miR-499	22 / 20	29 ± 10 / 30 ± 9	18.2 / 20	PBMC	NA	NA	qRT-PCR	NA	MCI	Healthy subjects
[48]	1478	miR-21	29 / 20	AMC: 32±10	AMC: 34,5	PBMC	NA	NA	qRT-PCR	NA	MCI	Healthy subjects

				Control: 30±9	Control: 25							
[49]	1485	miR-21	55 / 22	30 ± 12 / 30 ± 9	27,3 / 25	PBMC	NA	NA	qRT-PCR	NA	MCI	Healthy subjects
[50]	1497	miR-208b	55 / 22	30 ± 12 / 30 ± 9	27,3 / 25	PBMC	NA	NA	qRT-PCR	NA	MCI	Healthy subjects
[51]	1542	let-7f-5p<br>miR-135b-5p<br>miR-155-5p<br>miR-190a-5p<br>miR-296-5p<br>miR-339-5p<br>miR-365a-3p<br>miR-422a<br>miR-489-3p<br>miR-520e<br>miR-532-5p<br>miR-590-5p<br>miR-601<br>miR-660-5p<br>miR-1290	CVB3 perseverance DCM: 7<br>CVB3 eliminates DCM: 6<br>Control: 6<br>Adenovirus DCM: 4	CVB3 perseverance DCM: 50,2 ± 7,3<br>CVB3 eliminates DCM: 45,2 ± 13,2<br>Control: 48 ± 12<br>Adenovirus DCM: 52 ± 9	CVB3 perseverance DCM: 28,6<br>CVB3 eliminates DCM: 16,7<br>Control: 50<br>Adenovirus DCM: 25	EMB	Microarray Affymetrix U133 plus 2.0	Applied Biosystems mirVana miRNA Isolation Kit	Microarray Affymetrix U133 plus 2.0	NA	InfDCM	Healthy subjects
[52]	2179	miR-221<br>miR-222	18 / 34	NA	NA	Heart tissue	NA	miRVANA extraction kit (Ambion)	qRT-PCR	NA	MCI	DCM
[53]	2293	miR-10b-5p<br>miR-362-5p	6 / 3	53.5±14.8 / 53.7±7.37	50 / 33	Serum	qRT-PCR Panel	miRCURY RNA Isolation Biofluids Kit	qRT-PCR, miRCURY Locked Nucleic Acid and Universal RT microRNA PCR system	NA	Ni-IDCM	Healthy subjects
[54]	2323	miR-21<br>miR-34a<br>miR-423<br>miR-208a<br>miR-499a-5p	DCM: 9<br>ICM: 7<br>Control: 10	NA	NA	Plasma	NA	NA	qRT-PCR	NA	Ni-IDCM	Healthy subjects, Ischemic Cardiomyopathy
[55]	2540	miR-29<br>miR-122<br>miR-144<br>miR-206<br>miR-208a<br>miR-223<br>miR-499<br>miR-451-DICER1<br>miR-486<br>miR-3940	DCM: 6<br>ICM: 7<br>Control: 3<br>Control fetus: 3	NA	NA	Heart tissue	NA	Guanidinium thiocyanate-phenol-chloroform method	Illumina sequencing	NA	Ni-IDCM	Healthy subjects, healthy fetus, Ischemic Cardiomiopathy

Orange marked studies performed histological assessment of EMB or heart tissue samples.

1. Blanco-Domínguez, R.; Sánchez-Díaz, R.; de la Fuente, H.; Jiménez-Borreguero, L.J.; Matesanz-Marín, A.; Relaño, M.; Jiménez-Alejandre, R.; Linillos-Pradillo, B.; Tsilingiri, K.; Martín-Mariscal, M.L.; et al. A Novel Circulating MicroRNA for the Detection of Acute Myocarditis. *New England Journal of Medicine* **2021**, *384*, 2014-2027, doi:10.1056/NEJMoa2003608.
2. Brundin, M.; Wågsäter, D.; Alehagen, U.; Carlhäll, C.-J. Circulating microRNA-29-5p can add to the discrimination between dilated cardiomyopathy and ischaemic heart disease. *ESC Heart Failure* **2021**, *8*, 3865-3874, doi:https://doi.org/10.1002/ehf2.13458.
3. Calderon-Dominguez, M.; Belmonte, T.; Quezada-Feijoo, M.; Ramos, M.; Calderon-Dominguez, J.; Campuzano, O.; Mangas, A.; Toro, R. Plasma microrna expression profile for reduced ejection fraction in dilated cardiomyopathy. *Scientific Reports* **2021**, *11*, 7517, doi:10.1038/s41598-021-87086-1.
4. Ishiguro, T.; Hayashi, M.; Fujiwara, W.; Okumura, S.; Yoshinaga, M.; Yamada, R.; Ueda, S.; Ito, T.; Niwa, Y.; Miyazaki, A.; et al. Circulating miR-489 as a potential new biomarker for idiopathic dilated cardiomyopathy. *Fujita Medical Journal* **2021**, *7*, 18-22, doi:10.20407/fmj.2020-001.
5. Jiao, M.; You, H.-Z.; Yang, X.-Y.; Yuan, H.; Li, Y.-L.; Liu, W.-X.; Jin, M.; Du, J. Circulating microRNA signature for the diagnosis of childhood dilated cardiomyopathy. *Scientific Reports* **2018**, *8*, 724, doi:10.1038/s41598-017-19138-4.
6. Wang, H.; Chen, F.; Tong, J.; Li, Y.; Cai, J.; Wang, Y.; Li, P.; Hao, Y.; Tian, W.; Lv, Y.; et al. Circulating microRNAs as novel biomarkers for dilated cardiomyopathy. *Cardiol J* **2017**, *24*, 65-73, doi:10.5603/CJ.a2016.0097.
7. Chen, J.H.; He, J.; Zhou, R.; Zheng, N. [Expression and Significance of Circulating microRNA-29b in Adult Fulminant Myocarditis]. *Zhongguo Yi Xue Ke Xue Yuan Xue Bao* **2022**, *44*, 102-109, doi:10.3881/j.issn.1000-503X.14043.
8. Aleshcheva, G.; Pietsch, H.; Escher, F.; Schultheiss, H.-P. MicroRNA profiling as a novel diagnostic tool for identification of patients with inflammatory and/or virally induced cardiomyopathies. *ESC Heart Failure* **2021**, *8*, 408-422, doi:https://doi.org/10.1002/ehf2.13090.
9. Enes Coşkun, M.; Kervancıoğlu, M.; Öztuzcu, S.; Yılmaz Coşkun, F.; Ergün, S.; Başpınar, O.; Kılınç, M.; Temel, L.; Coşkun, M.Y. Plasma microRNA profiling of children with idiopathic dilated cardiomyopathy. *Biomarkers* **2016**, *21*, 56-61, doi:10.3109/1354750X.2015.1118533.
10. Yang, H.; Shan, L.; Gao, Y.; Li, L.; Xu, G.; Wang, B.; Yin, X.; Gao, C.; Liu, J.; Yang, W. MicroRNA-181b Serves as a Circulating Biomarker and Regulates Inflammation in Heart Failure. *Disease Markers* **2021**, *2021*, 4572282, doi:10.1155/2021/4572282.
11. Corsten, M.F.; Dennert, R.; Jochems, S.; Kuznetsova, T.; Devaux, Y.; Hofstra, L.; Wagner, D.R.; Staessen, J.A.; Heymans, S.; Schroen, B. Circulating MicroRNA-208b and MicroRNA-499 Reflect Myocardial Damage in Cardiovascular Disease. *Circulation: Cardiovascular Genetics* **2010**, *3*, 499-506, doi:10.1161/CIRCGENETICS.110.957415.

12. Onrat, S.T.; Onrat, E.; Ercan Onay, E.; Yalım, Z.; Avşar, A. The Genetic Determination of the Differentiation Between Ischemic Dilated Cardiomyopathy and Idiopathic Dilated Cardiomyopathy. *Genetic Testing and Molecular Biomarkers* **2018**, *22*, 644-651, doi:10.1089/gtmb.2018.0188.
13. Zhang, Y.; Sun, L.; Sun, H.; Yu, Z.; Liu, X.; Luo, X.; Li, C.; Sun, D.; Li, T. MicroRNA-381 protects myocardial cell function in children and mice with viral myocarditis via targeting cyclooxygenase-2 expression. *Exp Ther Med* **2018**, *15*, 5510-5516, doi:10.3892/etm.2018.6082.
14. Li, H.; Xie, Y.; Liu, Y.; Qi, Y.; Tang, C.; Li, X.; Zuo, K.; Sun, D.; Shen, Y.; Pang, D.; et al. Alteration in microRNA-25 expression regulate cardiac function via renin secretion. *Experimental Cell Research* **2018**, *365*, 119-128, doi:https://doi.org/10.1016/j.yexcr.2018.02.029.
15. Obradovic, D.; Rommel, K.-P.; Blazek, S.; Klingel, K.; Gutberlet, M.; Lücke, C.; Büttner, P.; Thiele, H.; Adams, V.; Lurz, P.; et al. The potential role of plasma miR-155 and miR-206 as circulatory biomarkers in inflammatory cardiomyopathy. *ESC Heart Failure* **2021**, *8*, 1850-1860, doi:https://doi.org/10.1002/ehf2.13304.
16. Rubiś, P.; Totoń-Żurańska, J.; Wiśniowska-Śmiałek, S.; Dziewięcka, E.; Kołton-Wróż, M.; Wołkow, P.; Pitera, E.; Rudnicka-Sosin, L.; Garlitski, A.C.; Gackowski, A.; et al. The relationship between myocardial fibrosis and myocardial microRNAs in dilated cardiomyopathy: A link between mir-133a and cardiovascular events. *Journal of Cellular and Molecular Medicine* **2018**, *22*, 2514-2517, doi:https://doi.org/10.1111/jcmm.13535.
17. Marketou, M.; Kontaraki, J.; Patrianakos, A.; Kochiadakis, G.; Anastasiou, I.; Fragkiadakis, K.; Plevritaki, A.; Papadaki, S.T.; Chlouverakis, G.; Parthenakis, F. Peripheral Blood MicroRNAs as Potential Biomarkers of Myocardial Damage in Acute Viral Myocarditis. *Genes* **2021**, *12*, doi:10.3390/genes12030420.
18. Fan, K.L.; Zhang, H.F.; Shen, J.; Zhang, Q.; Li, X.L. Circulating microRNAs levels in Chinese heart failure patients caused by dilated cardiomyopathy. *Indian Heart J* **2013**, *65*, 12-16, doi:10.1016/j.ihj.2012.12.022.
19. Yu, M.; Liang, W.; Xie, Y.; Long, Q.; Cheng, X.; Liao, Y.-H.; Yuan, J. Circulating miR-185 might be a novel biomarker for clinical outcome in patients with dilated cardiomyopathy. *Scientific Reports* **2016**, *6*, 33580, doi:10.1038/srep33580.
20. Xia, K.; Zhang, Y.; Sun, D. miR-217 and miR-543 downregulation mitigates inflammatory response and myocardial injury in children with viral myocarditis by regulating the SIRT1/AMPK/NF-κB signaling pathway. *Int J Mol Med* **2020**, *45*, 634-646, doi:10.3892/ijmm.2019.4442.
21. Zhang, Y.; Li, X.; Wang, D.; Jiang, X.; Zhang, M.; Lv, K. Serum exosome microRNA panel as a noninvasive biomarker for molecular diagnosis of fulminant myocarditis. *Molecular Therapy - Methods & Clinical Development* **2021**, *20*, 142-151, doi:https://doi.org/10.1016/j.omtm.2020.11.006.
22. Zhang, B.Y.; Zhao, Z.; Jin, Z. Expression of miR-98 in myocarditis and its influence on transcription of the FAS/FASL gene pair. *Genet Mol Res* **2016**, *15*, doi:10.4238/gmr.15027627.
23. Gumus, G.; Giray, D.; Bobusoglu, O.; Tamer, L.; Karpuz, D.; Hallioglu, O. MicroRNA values in children with rheumatic carditis: a preliminary study. *Rheumatology International* **2018**, *38*, 1199-1205, doi:10.1007/s00296-018-4069-2.
24. Corsten, M.F.; Papageorgiou, A.; Verhesen, W.; Carai, P.; Lindow, M.; Obad, S.; Summer, G.; Coort, S.L.M.; Hazebroek, M.; van Leeuwen, R.; et al. MicroRNA Profiling Identifies MicroRNA-155 as an Adverse Mediator of Cardiac Injury and Dysfunction During Acute Viral Myocarditis. *Circulation Research* **2012**, *111*, 415-425, doi:10.1161/CIRCRESAHA.112.267443.
25. Woulfe, K.C.; Siomos, A.K.; Nguyen, H.; SooHoo, M.; Galambos, C.; Stauffer, B.L.; Sucharov, C.; Miyamoto, S. Fibrosis and Fibrotic Gene Expression in Pediatric and Adult Patients With Idiopathic Dilated Cardiomyopathy. *Journal of Cardiac Failure* **2017**, *23*, 314-324, doi:https://doi.org/10.1016/j.cardfail.2016.11.006.
26. Nie, X.; He, M.; Wang, J.; Chen, P.; Wang, F.; Lai, J.; Li, C.; Yu, T.; Zuo, H.; Cui, G.; et al. Circulating miR-4763-3p Is a Novel Potential Biomarker Candidate for Human Adult Fulminant Myocarditis. *Molecular Therapy - Methods & Clinical Development* **2020**, *17*, 1079-1087, doi:https://doi.org/10.1016/j.omtm.2020.05.005.
27. Voellenkle, C.; van Rooij, J.; Cappuzzello, C.; Greco, S.; Arcelli, D.; Di Vito, L.; Melillo, G.; Rigolini, R.; Costa, E.; Crea, F.; et al. MicroRNA signatures in peripheral blood mononuclear cells of chronic heart failure patients. *Physiological Genomics* **2010**, *42*, 420-426, doi:10.1152/physiolgenomics.00211.2009.
28. Fan, K.L.; Li, M.F.; Cui, F.; Feng, F.; Kong, L.; Zhang, F.H.; Hao, H.; Yin, M.X.; Liu, Y. Altered exosomal miR-181d and miR-30a related to the pathogenesis of CVB3 induced myocarditis by targeting SOCS3. *Eur Rev Med Pharmacol Sci* **2019**, *23*, 2208-2215, doi:10.26355/eurrev_201903_17268.
29. Glezeva, N.; Moran, B.; Collier, P.; Moravec, C.S.; Phelan, D.; Donnellan, E.; Russell-Hallinan, A.; O'Connor, D.P.; Gallagher, W.M.; Gallagher, J.; et al. Targeted DNA Methylation Profiling of Human Cardiac Tissue Reveals Novel Epigenetic Traits and Gene Deregulation Across Different Heart Failure Patient Subtypes. *Circ Heart Fail* **2019**, *12*, e005765, doi:10.1161/circheartfailure.118.005765.
30. Zhang, H.B.; Li, R.C.; Xu, M.; Xu, S.M.; Lai, Y.S.; Wu, H.D.; Xie, X.J.; Gao, W.; Ye, H.; Zhang, Y.Y.; et al. Ultrastructural uncoupling between T-tubules and sarcoplasmic reticulum in human heart failure. *Cardiovasc Res* **2013**, *98*, 269-276, doi:10.1093/cvr/cvt030.
31. Wu, T.; Chen, Y.; Du, Y.; Tao, J.; Zhou, Z.; Yang, Z. Serum Exosomal MiR-92b-5p as a Potential Biomarker for Acute Heart Failure Caused by Dilated Cardiomyopathy. *Cell Physiol Biochem* **2018**, *46*, 1939-1950, doi:10.1159/000489383.
32. Besler, C.; Urban, D.; Watzka, S.; Lang, D.; Rommel, K.P.; Kandolf, R.; Klingel, K.; Thiele, H.; Linke, A.; Schuler, G.; et al. Endomyocardial miR-133a levels correlate with myocardial inflammation, improved left ventricular function, and clinical outcome in patients with inflammatory cardiomyopathy. *Eur J Heart Fail* **2016**, *18*, 1442-1451, doi:10.1002/ejhf.579.
33. Ikeda, S.; Kong, S.W.; Lu, J.; Bisping, E.; Zhang, H.; Allen, P.D.; Golub, T.R.; Pieske, B.; Pu, W.T. Altered microRNA expression in human heart disease. *Physiological Genomics* **2007**, *31*, 367-373, doi:10.1152/physiolgenomics.00144.2007.
34. Chen, Z.G.; Liu, H.; Zhang, J.B.; Zhang, S.L.; Zhao, L.H.; Liang, W.Q. Upregulated microRNA-214 enhances cardiac injury by targeting ITCH during coxsackievirus infection. *Mol Med Rep* **2015**, *12*, 1258-1264, doi:10.3892/mmr.2015.3539.
35. Baumgarten, A.; Bang, C.; Tschirner, A.; Engelmann, A.; Adams, V.; von Haehling, S.; Doehner, W.; Pregla, R.; Anker, M.S.; Blecharz, K.; et al. TWIST1 regulates the activity of ubiquitin proteasome system via the miR-199/214 cluster in human end-stage dilated cardiomyopathy. *International Journal of Cardiology* **2013**, *168*, 1447-1452, doi:https://doi.org/10.1016/j.ijcard.2012.12.094.
36. Wang, D.; Li, T.; Cui, H.; Zhang, Y. Analysis of the Indicating Value of Cardiac Troponin I, Tumor Necrosis Factor-α, Interleukin-18, Mir-1 and Mir-146b for Viral Myocarditis among Children. *Cellular Physiology and Biochemistry* **2016**, *40*, 1325-1333, doi:10.1159/000453185.
37. Bao, J.L.; Lin, L. MiR-155 and miR-148a reduce cardiac injury by inhibiting NF-κB pathway during acute viral myocarditis. *Eur Rev Med Pharmacol Sci* **2014**, *18*, 2349-2356.
38. Naga Prasad, S.V.; Gupta, M.K.; Duan, Z.-H.; Surampudi, V.S.K.; Liu, C.-G.; Kotwal, A.; Moravec, C.S.; Starling, R.C.; Perez, D.M.; Sen, S.; et al. A unique microRNA profile in end-stage heart failure indicates alterations in specific cardiovascular signaling networks. *PLOS ONE* **2017**, *12*, e0170456, doi:10.1371/journal.pone.0170456.
39. Hailu, F.T.; Karimpour-Fard, A.; Toni, L.S.; Bristow, M.R.; Miyamoto, S.D.; Stauffer, B.L.; Sucharov, C.C. Integrated analysis of miRNA–mRNA interaction in pediatric dilated cardiomyopathy. *Pediatric Research* **2022**, *92*, 98-108, doi:10.1038/s41390-021-01548-w.
40. Yan, M.; Wang, J.; Wang, S.; Zhang, Y.; Liu, L.; Zhao, H. Expression Levels of MicroRNA-146b and Anti-Cardiac Troponin I in Serum of Children with Viral Myocarditis and Their Clinical Significance. *Iran J Public Health* **2021**, *50*, 510-519, doi:10.18502/ijph.v50i3.5592.
41. JIAO, M.; YOU, H.; WANG, Z.; GU, Y.; WANG, X.; LIANG, Y.; XIAO, Y.; JIN, M. Diagnosis of dilated cardiomyopathy in children based on microRNA sequencing technology [基于微小RNA测序技术的儿童扩张型心肌病的诊断学研究]. *Chinese J. Appl. Clin. Pediatr.* **2020**, *24*, 982-987.
42. Zeng, Z.; Wang, K.; Li, Y.; Xia, N.; Nie, S.; Lv, B.; Zhang, M.; Tu, X.; Li, Q.; Tang, T.; et al. Down-regulation of microRNA-451a facilitates the activation and proliferation of CD4⁺ T cells by targeting Myc in patients with dilated cardiomyopathy. *Journal of Biological Chemistry* **2017**, *292*, 6004-6013, doi:10.1074/jbc.M116.765107.
43. Baán, J.A.; Varga, Z.V.; Leszek, P.; Kuśmierczyk, M.; Baranyai, T.; Dux, L.; Ferdinandy, P.; Braun, T.; Mendler, L. Myostatin and IGF-I signaling in end-stage human heart failure: a qRT-PCR study. *Journal of Translational Medicine* **2015**, *13*, 1, doi:10.1186/s12967-014-0365-0.

44. Tao, L.; Yang, L.; Huang, X.; Hua, F.; Yang, X. Reconstruction and Analysis of the lncRNA-miRNA-mRNA Network Based on Competitive Endogenous RNA Reveal Functional lncRNAs in Dilated Cardiomyopathy. *Front Genet* **2019**, *10*, 1149, doi:10.3389/fgene.2019.01149.
45. Besler, C.; Urban, D.; Watzka, S.; Klingel, K.; Kandolf, R.; Schuler, G.; Adams, V.; Lurz, P. Abstract 17607: MicroRNA Expression Profiles in Endomyocardial Biopsies From Patients With Myocarditis - Association With Left Ventricular Function and Clinical Events. *Circulation* **2015**, *132*, A17607-A17607, doi:doi:10.1161/circ.132.suppl_3.17607.
46. Satoh, M.; Minami, Y.; Takahashi, Y.; Tabuchi, T.; Nakamura, M. Expression of microRNA-208 is associated with adverse clinical outcomes in human dilated cardiomyopathy. *J Card Fail* **2010**, *16*, 404-410, doi:10.1016/j.cardfail.2010.01.002.
47. Marketou, M.; Kontaraki, J.; Konstantinou, J.; Parthenakis, F.; Nakou, H.; Lempidakis, D.; Vernardos, M.; Fragkiadakis, K.; Loulakakis, M.; Theodosaki, O.; et al. P2579Differential microRNA gene expression levels in patients with acute myocarditis. *European Heart Journal* **2017**, *38*, ehx502.P2579, doi:10.1093/eurheartj/ehx502.P2579.
48. Marketou, M.; Kontaraki, J.; Konstantinou, J.; Parthenakis, F.; Patrianakos, A.; Nakou, H.; Lempidakis, D.; Vernardos, M.; Fragkiadakis, K.; Loulakakis, M.; et al. P2573MicroRNA-21 gene expression levels in peripheral monocytes reflect myocardial damage in acute myocarditis. *European Heart Journal* **2017**, *38*, ehx502.P2573, doi:10.1093/eurheartj/ehx502.P2573.
49. Marketou, M.; Kontaraki, J.; Konstantinou, J.; Maragkoudakis, S.; Plevritaki, A.; Lempidakis, D.; Vougia, D.; Fragiadakis, K.; Kassotakis, S.; Theodosaki, O.; et al. P5562Increased microRNA-21 gene expression levels as a biomarker of myocardial damage in acute myocarditis. *European Heart Journal* **2019**, *40*, ehz746.0506, doi:10.1093/eurheartj/ehz746.0506.
50. Marketou, M.; Kontaraki, J.; Fragiadakis, K.; Konstantinou, J.; Maragkoudakis, S.; Lempidakis, D.; Plevritaki, A.; Plataki, M.; Papadaki, S.; Vougia, D.; et al. MicroRNA-208b gene expression levels as a biomarkers of left ventricular dysfunction in patients with acute myocarditis. *European Heart Journal* **2020**, *41*, doi:10.1093/ehjci/ehaa946.2056.
51. Kuehl, U.; Lassner, D.; Gast, M.; Stroux, A.; Rohde, M.; Siegismund, C.; Wang, X.; Escher, F.; Gross, M.; Skurk, C.; et al. Differential Cardiac MicroRNA Expression Predicts the Clinical Course in Human Enterovirus Cardiomyopathy. *Circ Heart Fail* **2015**, *8*, 605-618, doi:10.1161/circheartfailure.114.001475.
52. Heggermont, W.A.; Delrue, L.; Dierckx, R.; Dierickx, K.; Verstreken; Goethals, M.; Bartunek, J.; Vanderheyden, M. The MiR-221/-222 cluster is elevated both in biopsies of patients with myocarditis and idiopathic non-ischemic cardiomyopathy but fails to discriminate between both pathologies. *European Journal of Heart Failure* **2018**, *28*, 23.
53. The 4th Joint EFLM-UEMS Congress "Laboratory Medicine at the Clinical Interface" Warsaw, Poland, 21th–24th September, 2016. *Clinical Chemistry and Laboratory Medicine (CCLM)* **2016**, *54*, eA213-eA366, doi:doi:10.1515/cclm-2016-0657.
54. Zaseeva, A.V.; Zhirov, I.V.; Tereschenko, S.N.; Scvortcov, A.A.; Masenko, V.P.; Kochetov, A.G.; Gimadiev, R.R.; Abramov; Lyang, O.V. The role of circulating miR-21, miR-34a, miR-423, miR-208a and miR-499a in ischemic and dilated cardiomyopathy. *European Journal of Heart Failure* **2016**, *18*, 492.
55. Akat, K.M.; Mihailovic, A.; Williams, Z.; Brown, M.; Morozov, P.; Takayama, H.; Drosatos, K.; Tuschl, T.; Schulze, P.C. Abstract 10918: High-Throughput Sequencing Analysis of microRNA Profile Dynamics in Patients with Advanced Heart Failure Undergoing Ventricular Assist Device Placement in Comparison to Normal Adult and Fetal Cardiac Expression. *Circulation* **2011**, *124*, A10918-A10918, doi:10.1161/circ.124.suppl_21.A10918.
